# Protocol for whole-tissue immunolabeling, optical clearing, and lightsheet imaging of c-Fos protein expression in unsectioned mouse brains

**DOI:** 10.1016/j.xpro.2025.103868

**Published:** 2025-05-31

**Authors:** Koukou Fu, Jing Yang

**Affiliations:** 1 State Key Laboratory of Membrane Biology, School of Life Sciences, Center for Life Sciences, IDG/McGovern Institute for Brain Research, Peking University Third Hospital Cancer Center, Peking University, Beijing 100871, China; 2 Peking Union Medical College Hospital, Beijing 100730, China

**Keywords:** Microscopy, Neuroscience, Biotechnology and bioengineering

## Abstract

The c-Fos protein has been broadly utilized as a marker of neuronal activity, and conventional immunohistochemistry to determine its expression relies on tissue sections. Here, we present a protocol to visualize the endogenous c-Fos protein in intact, unsectioned mouse brains responding to specific stimuli based on the immunolabeling-enabled three-dimensional imaging of solvent-cleared organs (iDISCO) method. We describe steps for tissue harvesting, fixation, decolorization, and permeabilization followed by whole-tissue anti-c-Fos immunolabeling. We then detail procedures for tissue embedding and optical clearing for imaging by lightsheet microscopy.

For complete details on the use and execution of this protocol, please refer to Chen et al.^1^

## Highlights

• Steps for immunolabeling of the endogenous c-Fos protein in unsectioned mouse brains
• Instructions for optical clearing for lightsheet imaging of c-Fos protein expression
• Guide to visualizing c-Fos expression in mouse brains responding to specific stimuli

## Abstract (editor highlights)

Publisher’s note: Undertaking any experimental protocol requires adherence to local institutional guidelines for laboratory safety and ethics.

## Graphical abstract

**Figure:**
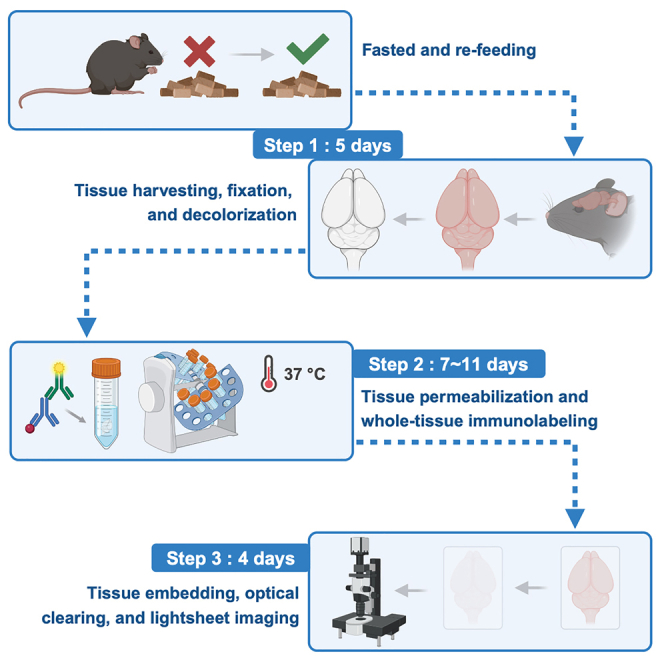

## Before you begin

### Institutional permission

All animal experiments must be approved by the Institutional Animal Care and Use Committee (IACUC) at your research institution. The mouse procedures described in this protocol were performed in compliance with the protocol approved by the Institutional Animal Care and Use Committee (IACUC) of Peking University.

### Preparation for tissue harvesting, fixation, and decolorization

**Timing: 1 h (for steps 1 and 2)**

**Timing: 1 h (for steps 3 and 4)**

**Timing: 30 min (for steps 5 and 6)**

1. Prepare Formaldehyde Stock Solution (4%).
  a. Add 40 g of paraformaldehyde powder to 950 mL ddH_2_O preheated to 60°C.
  b. Add 5 M NaOH dropwise with gentle stirring until paraformaldehyde powder is completely dissolved.
  c. Adjust the final volume to 1000 mL with ddH_2_O.

***Note:*** The pH of the solution should be 7.4. Adjust if needed.

***Note:*** This can be done 1 week in advance. Store at 4°C.

**CRITICAL:** Handle paraformaldehyde in a chemical hood and dispose it according to local health and safety regulations.

2. Prepare Heparin Stock Solution (10 mg/mL).
  a. Add 1 g of heparin sodium powder into 100 mL ddH_2_O.
  b. Rotate until heparin is completely dissolved.
  c. Filter the solution through a 0.22-μm syringe filter.

***Note:*** This can be done 1 week in advance. Store at 4°C.

3. Prepare Perfusion Buffer A and Perfusion Buffer B.

***Note:*** Please refer to the “Materials and equipment” section for the detailed compositions of Perfusion Buffer A and Perfusion Buffer B.

***Note:*** All the Perfusion Buffers must be freshly prepared on the day of use for the optimal quality of tissue fixation.

***Note:*** Prewarm the Perfusion Buffers to 37°C before tissue harvesting.

**CRITICAL:** Handle all the Perfusion Buffers in a chemical hood and dispose them according to local health and safety regulations.

4. Prepare Fixation Buffer.

***Note:*** Please refer to the “Materials and equipment” section for the detailed compositions of Fixation Buffer.

***Note:*** Fixation Buffer must be freshly prepared on the day of use for the optimal quality of tissue fixation.

**CRITICAL:** Handle Fixation Buffer in a chemical hood and dispose it according to local health and safety regulations.

5. Prepare 20%, 40%, 60%, 80% (v/v) methanol in ddH_2_O.

***Note:*** This can be done 1 week in advance. Store at 22°C–25°C.

**CRITICAL:** Handle methanol in a chemical hood and dispose the methanol-containing solutions according to local health and safety regulations.

**CRITICAL:** Use glass containers (i.e., bottles and vials) with polypropylene caps and styrene–butadiene rubber liners for handling organic solvents (i.e., methanol, dichloromethane, and dibenzyl ether) throughout this protocol. The organic solvents, especially dichloromethane, can easily erode common plastic labware (e.g., pipettes, tubes, and dishes).

6. Prepare Decolorization Solution.

***Note:*** Please refer to the “Materials and equipment” section for the detailed compositions of Decolorization Solution.

***Note:*** This can be done 1 day in advance. Precool the Decolorization Solution to 4°C before use.

**CRITICAL:** Handle methanol and H_2_O_2_ in a chemical hood and dispose them according to local health and safety regulations.

### Preparation for tissue permeabilization and whole-tissue immunolabeling

**Timing: 90 min (for steps 7, 8, 9, 10, and 11)**

**Timing: 10 min (for step 12)**

**Timing: 20 min (for step 13)**

**Timing: 10 min (for step 14)**

7. Prepare EDTA-Na Stock Solution (0.5 M).
  a. Add 73 g of EDTA to 480 mL ddH_2_O.
  b. Add 5 M NaOH dropwise with gentle stirring until EDTA powder is completely dissolved.
  c. Adjust the final volume to 500 mL with ddH_2_O.

***Note:*** The pH of the solution should be 8.0. Adjust if needed.

***Note:*** This can be done 1 week in advance. Store at 4°C.

8. Prepare Triton X-100 Stock Solution (10% (w/v)).
  a. Add 5 g of Triton X-100 to 40 mL ddH_2_O.
  b. Continuously stir until solution becomes visually homogeneous.
  c. Adjust the final volume to 50 mL with ddH_2_O.

***Note:*** This can be done 1 week in advance. Store at 4°C.

9. Prepare Deoxycholate Stock Solution (5% (w/v)).
  a. Add 5 g of deoxycholate powder to 90 mL ddH_2_O.
  b. Adjust the final volume to 100 mL with ddH_2_O.

***Note:*** This can be done 1 week in advance. Store at 4°C.

10. Prepare Tween 20 Stock Solution (10% (w/v)).
  a. Add 10 g of Tween 20 to 85 mL ddH_2_O.
  b. Continuously stir until solution becomes visually homogeneous.
  c. Adjust the final volume to 100 mL with ddH_2_O.

***Note:*** This can be done 1 week in advance. Store at 4°C.

11. Prepare Sodium Azide Stock Solution (10% (w/v)).
  a. Add 1 g of sodium azide to 8 mL ddH_2_O.
  b. Adjust the final volume to 10 mL with ddH_2_O.

***Note:*** This can be done 1 week in advance. Store at 4°C.

12. Prepare Permeabilization Buffer.

***Note:*** Please refer to the “Materials and equipment” section for the detailed compositions of Permeabilization Buffer.

***Note:*** Filter the buffer through a 0.22-μm syringe filter. This can be done 1 week in advance. Store at 22°C–25°C.

13. Prepare Immunolabeling Buffer.

***Note:*** Please refer to the “Materials and equipment” section for the detailed compositions of Immunolabeling Buffer.

***Note:*** Filter the buffer through a 0.22-μm syringe filter after diluting the primary or secondary antibody. This can be done 1 day in advance. Store at 4°C.

14. Prepare Washing Buffer.

***Note:*** Please refer to the “Materials and equipment” section for the detailed compositions of Washing Buffer.

***Note:*** Filter the buffer through a 0.22-μm syringe filter. This can be done 1 week in advance. Store at 22°C–25°C.

### Preparation for tissue embedding, optical clearing, and lightsheet imaging

**Timing: 15 min (for step 15)**

**Timing: 10 min (for step 16)**

**Timing: 10 min (for step 17)**

15. Prepare 0.8% agarose.
  a. Add 0.8 g of agarose powder to 100 mL 1× PBS.
  b. Heat the mixture in a microwave oven until agarose powder is completely dissolved.

***Note:*** 0.8% agarose must be made freshly and cooled down to approximately 50°C before tissue embedding.

16. Prepare a mixture of dichloromethane and methanol (v:v=2:1).

***Note:*** This must be done on the day of use.

**CRITICAL:** Handle dichloromethane and methanol in a chemical hood and dispose them according to local health and safety regulations.

**CRITICAL:** Prepare in glass bottles with polypropylene caps and styrene–butadiene rubber liners.

17. Fill the imaging chamber with 100% dibenzyl-ether.

***Note:*** This must be done on the day of use.

**CRITICAL:** Handle dibenzyl-ether in a chemical hood and dispose it according to local health and safety regulations.

## Key resources table

**Table:** 

REAGENT or RESOURCE	SOURCE	IDENTIFIER
**Antibodies**		

Rabbit anti-c-Fos (1:500 dilution)	Synaptic Systems	Cat#226008; RRID: AB_2891278
Donkey anti-rabbit IgG (H+L), Alexa Fluor Plus 647 (1:1,000 dilution)	Thermo Fisher Scientific	Cat#A32795; RRID: AB_2762835

C**hemicals, peptides, and recombinant proteins**		

Phosphate-buffered saline (PBS), 10 ×	Sigma-Aldrich	Cat#P7059
Heparin sodium salt	MedChemExpress	Cat#HY-17567A
Paraformaldehyde	Sigma-Aldrich	Cat#158172
Sodium hydroxide solution, 5.0 M	Sigma-Aldrich	Cat#S8263
Hydrogen peroxide (H_2_O_2_), 30% (w/w)	Sigma-Aldrich	Cat#H1009
Ethylenediaminetetraacetic acid (EDTA)	Sigma-Aldrich	Cat#E6758
Triton X-100	Sigma-Aldrich	Cat#T8787
Sodium deoxycholate	Sigma-Aldrich	Cat#D6750
Tween 20	Sigma-Aldrich	Cat#655205
Sodium azide	Sigma-Aldrich	Cat#SRE0046
Dimethyl sulfoxide (DMSO)	Sigma-Aldrich	Cat#D4540
Methanol	TGREAG	Cat#104028
Dichloromethane	TGREAG	Cat#102045
Dibenzyl ether	Macklin	Cat#D807110
Agarose	Tsingke	Cat#TSJ001
Normal donkey serum	Jackson ImmunoResearch	Cat#017-000-121; RRID: AB_2337258

**Experimental models: Organisms/strains**		

Mouse: C57BL/6 wild-type, 8 weeks old, male	GemPharmatech	Cat#N000013

**Software and algorithms**		

Imaris	Oxford Instruments	https://imaris.oxinst.com/packages

**Other**		

Ultramicroscope II	LaVision BioTec	N/A

## Materials and equipment

**Table:** Perfusion buffer A

Reagent	Final concentration	Amount
10 × PBS	1 ×	50 mL
10 mg/mL Heparin	50 μg/mL	2.5 mL
ddH_2_O	N/A	447.5 mL
**Total Volume**	**N/A**	**500 mL**

Prepare fresh on the day of use. One-time use.

**Table:** Perfusion buffer B

Reagent	Final concentration	Amount
10 × PBS	1 ×	50 mL
10 mg/mL Heparin	50 μg/mL	2.5 mL
4% (w/v) Formaldehyde	1% (w/v)	125 mL
ddH_2_O	N/A	322.5 mL
**Total Volume**	**N/A**	**500 mL**

Prepare fresh on the day of use. One-time use.

**Table:** Fixation buffer

Reagent	Final concentration	Amount
10 × PBS	1 ×	50 mL
4% (w/v) Formaldehyde	1% (w/v)	125 mL
ddH_2_O	N/A	325 mL
**Total Volume**	**N/A**	**500 mL**

Prepare fresh on the day of use. One-time use.

**Table:** Decolorization solution

Reagent	Final concentration	Amount
Methanol	90% (v/v)	450 mL
30% (v/v) H_2_O_2_	3% (v/v)	50 mL
**Total Volume**	**N/A**	**500 mL**

Store at 4°C for up to 1 day.

**Table:** Permeabilization buffer

Reagent	Final concentration	Amount
10 × PBS	1 ×	50 mL
10% (w/v) Triton X-100	0.2% (v/v)	10 mL
5% (w/v) Deoxycholate	0.1% (w/v)	10 mL
DMSO	10% (v/v)	50 mL
0.5 M EDTA-Na (pH 8.0)	10 mM	10 mL
10% (w/v) Sodium azide	0.05% (w/v)	2.5 mL
ddH_2_O	N/A	367.5 mL
**Total Volume**	**N/A**	**500 mL**

Filter and store at 22∼25°C for up to 1 week.

**Table:** Immunolabeling buffer

Reagent	Final concentration	Amount
10 × PBS	1 ×	50 mL
10% (w/v) Tween 20	0.2%	10 mL
DMSO	5% (v/v)	25 mL
0.5 M EDTA-Na (pH 8.0)	10 mM	10 mL
10 mg/mL Heparin	10 μg/mL	0.5 mL
Normal donkey serum	5% (v/v)	25 mL
10% (w/v) Sodium azide	0.05% (w/v)	2.5 mL
ddH_2_O	N/A	377 mL
**Total Volume**	**N/A**	**500 mL**

Filter and store at 4°C for up to 1 day.

**Table:** Washing buffer

Reagent	Final concentration	Amount
10 × PBS	1 ×	100 mL
10% (w/v) Tween 20	0.2%	20 mL
0.5 M EDTA-Na (pH 8.0)	10 mM	20 mL
10 mg/mL Heparin	10 μg/mL	1 mL
ddH_2_O	N/A	859 mL
**Total Volume**	**N/A**	**1000 mL**

Filter and store at 22∼25°C for up to 1 week.

### Microscopy equipment

Perform lightsheet imaging on a LaVision Biotec Ultramicroscope II equipped with a 2× / NA0.5 objective covered with a 10 mm-working-distance dipping cap. Immerse a brain sample in the imaging chamber filled with 100% dibenzyl-ether. Utilize a SuperK FIANIUM FIU-15 supercontinuum white light laser (NKT Photonics) as the excitation source. This laser setup provides a total output power of 5.5 W. For imaging the Alexa Fluor Plus 647-conjugated secondary-antibody immunolabeling, set the excitation filter at 640/30 nm and the emission filter at 690/50 nm. Acquire an image stack with an Andor Neo sCMOS camera at 2560 × 2160 pixel resolution with the exposure time at 200 milliseconds for each optical section. For imaging at 1.26× (0.63× zoom) magnification, scan the brain sample by three combined lightsheets with a step size of 5 μm. For imaging at 2.5× (1.25× zoom) magnification, scan by three combined lightsheets with a step size of 2 μm. For imaging at 12.6× (6.3× zoom) magnification, scan by a single lightsheet (middle position) from the left side with a step size of 1 μm. Reconstruct the image stacks in Imaris and generate the representative 3D images shown in the figure by orthogonal projection.

## Step-by-step method details

### Tissue harvesting, fixation, and decolorization

**Timing: 5 days**

This section of the protocol details the steps for tissue harvesting, fixation, and decolorization following exposure of mice to specific stimuli. We present this protocol to visualize the c-Fos protein expression in the brain regions related to parasympathetic signals in response to food intake, as we recently reported.^1^ This is an example protocol, and time point may need to be adjusted according to each particular experiment.

***Note:*** Each tissue must be placed in a separate container. Perform all the incubation steps of brain samples with gentle rotation. Ensure the tissues are completely immersed.

1. Fast 8-week-old C57BL/6 wild-type male mice for 24 h. Subject the experimental group to the feeding condition for 3 h, while the control group remains fasted.
2. Perfuse the anesthetized mice transcardially with 25 mL Perfusion Buffer A, followed by 25 mL Perfusion Buffer B.
3. Dissect the whole brain of each mouse.
4. Postfix each brain with 25 mL of Fixation Buffer at 4°C for 16 h with gentle rotation.
5. Wash each brain with 25 mL of 1× PBS at 22°C–25°C for 1 h three times with gentle rotation.
6. Incubate each brain in a glass vial at 22°C–25°C with 15 mL of the methanol gradient: 20% methanol for 2 h, 40% methanol for 2 h, 60% methanol for 2 h, 80% methanol for 2 h, and 100% methanol for 2 h. Incubate with gentle rotation.

***Note:*** If the subsequent experimental steps are not to be performed immediately, the procedure can be paused at this stage. Store the tissues in 100% methanol at 4 °C for up to 6 months.

7. Cool the brains in 100% methanol at 4°C for 1 h.
8. Incubate each brain with 15 mL of Decolorization Solution in the same glass vial at 4°C for 48 h with gentle rotation.
9. Incubate each brain in the same glass vial at 22°C–25°C with 15 mL of the inverse methanol gradient: 100% methanol for 2 h, 80% methanol for 2 h, 60% methanol for 2 h, 40% methanol for 2 h, and 20% methanol for 2 h. Incubate with gentle rotation.

### Tissue permeabilization and whole-tissue immunolabeling

**Timing: 7–11 days**

This section of the protocol describes the whole-tissue immunolabeling of endogenous c-Fos protein in unsectioned mouse brains. The primary antibody used in this protocol is rabbit anti-c-Fos, and the secondary antibody is donkey anti-rabbit IgG (H+L) Alexa Fluor Plus 647. Information on the antibodies is in the key resources table.

***Note:*** Perform all the incubation steps with gentle rotation. Ensure the brain samples are completely immersed.

10. Incubate each brain with 25 mL of Permeabilization Buffer at 37°C for 24 h with gentle rotation.
11. Block each brain with 4 mL of Immunolabeling Buffer at 37°C for 24 h with gentle rotation.
12. Incubate each brain with 4 mL of the primary antibody diluted (final concentration of 2 μg/mL) in the Immunolabeling Buffer at 37°C for 48 h with gentle rotation.
13. Incubate each brain with 25 mL of Washing Buffer at 37°C for 24 h with gentle rotation. Change the fresh Washing Buffer every 6∼8 h.
14. Incubate the tissues with 4 mL of the secondary antibody diluted (final concentration of 2 μg/mL) in the Immunolabeling Buffer at 37°C for 48 h with gentle rotation.
15. Incubate each brain with 25 mL of Washing Buffer at 37°C for 48 h with gentle rotation. Change the fresh Washing Buffer every 8∼12 h.

***Note:*** After completing this step, the brain samples must be processed to the next steps immediately.

### Tissue embedding, optical clearing, and lightsheet imaging

**Timing: 4 days**

This section of the protocol includes the tissue embedding and optical clearing of mouse brain samples, which are finally subjected to lightsheet fluorescence imaging.

***Note:*** Perform all the incubation steps with gentle rotation. Ensure the tissues are completely immersed.

16. Examine the brain samples under a dissection microscope for any attached debris or dirt.

***Note:*** To avoid tissue desiccation, the brains can be briefly immersed in Washing Buffer before next step.

17. Place each brain in a separate 35-mm dish with the cerebral cortex facing upward. Embed in 0.8% agarose.

***Note:*** The 0.8% agarose should be cooled down to approximately 50°C.

18. Solidify the tissue blocks at 4°C for 30 min. Trim excess agarose surrounding the tissue block, ensuring that the remaining agarose does not exceed 1 mm from the tissue edges.
19. Incubate each embedded tissue block in a glass vial at 22°C–25°C with 15 mL of the methanol gradient: 20% methanol for 1 h three times; 40% methanol for 1 h, 60% methanol for 1 h, 80% methanol for 1 h, and 100% methanol for 1 h twice. Incubate with gentle rotation.
20. Incubate each tissue block in the same glass vial with a mixture (v:v = 2:1) of dichloromethane and methanol at 22°C–25°C for 2 h twice. Incubate with gentle rotation.
21. Incubate each tissue block in the same glass vial with dichloromethane at 22°C–25°C for 1 h three times. Incubate with gentle rotation.
22. Incubate each tissue block in the same glass vial with dibenzyl ether at 22°C–25°C for 12 h three times. Incubate with gentle rotation.

***Note:*** After completing the above steps, proceed to imaging as soon as possible. Samples can be stored in dibenzyl ether for up to 2 weeks.

**CRITICAL:** Handle dichloromethane, methanol, and dibenzyl ether in a chemical hood.

23. Mount the embedded brain sample into a sample holder, which is an accessory part of the LaVision Biotec Ultramicroscope II, with the brainstem facing upward.
24. Immerse a brain sample in the imaging chamber filled with 100% dibenzyl-ether.
25. Utilize a SuperK FIANIUM FIU-15 supercontinuum white light laser (NKT Photonics) as the excitation source. For imaging the Alexa Fluor Plus 647-conjugated secondary-antibody immunolabeling, set the excitation filter at 640/30 nm and the emission filter at 690/50 nm.

***Note:*** Select appropriate excitation and emission filters according to the particular fluorophore conjugated to a secondary antibody utilized for each experiment.

26. Acquire an image stack with an Andor Neo sCMOS camera at 2560 × 2160 pixel resolution with the exposure time at 200 ms for each optical section. For imaging at 1.26× (0.63× zoom) magnification, scan the brain sample by three combined lightsheets with a step size of 5 μm. For imaging at 2.5× (1.25× zoom) magnification, scan by three combined lightsheets with a step size of 2 μm. For imaging at 12.6× (6.3× zoom) magnification, scan by a single lightsheet (middle position) from the left side with a step size of 1 μm.
27. Reconstruct the image stacks in Imaris and generate the representative 3D images shown in the figure by orthogonal projection.

## Expected outcomes

The c-Fos protein has been utilized as a marker for neuronal activity for decades.^2^^,^^3^ This protocol is optimized based on the original technique of immunolabeling-enabled three-dimensional imaging of solvent-cleared organs (iDISCO) reported by us and colleagues.^4^^,^^5^ The procedure includes whole-tissue immunolabeling, optical clearing, and lightsheet 3D imaging of endogenous c-Fos protein expressions in intact, unsectioned mouse brains, enabling both visualization and quantification of c-Fos activities in different brain regions, as demonstrated in our previous publications.^1^^,^^4^^,^^5^ Compared to conventional immunohistochemistry methods that rely on brain tissue sections, this protocol offers a more comprehensive, accurate map of neuronal activities without the necessity of laborious, time-consuming reconstruction of tissue-section images.

**Figure 1:**
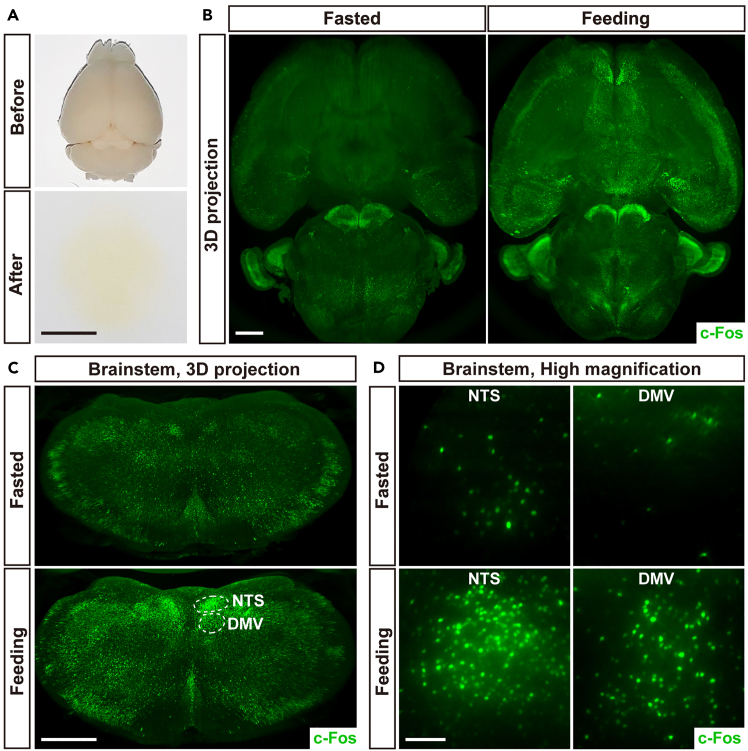
Whole-tissue immunolabeling, optical clearing, and lightsheet 3D imaging of endogenous c-Fos protein expression in unsectioned mouse brains (A) The unsectioned brain samples of adult C57BL/6 wild-type mice before (upper panel) and after (lower panel) being processed through whole-tissue immunolabeling and optical clearing. Scale bar, 5 mm. (B–D) Adult C57BL/6 wild-type mice were subjected to the fasting or feeding conditions. The unsectioned brain samples were processed through the protocol. (B) Representative 3D-projection images (transverse view) of the brain at 1.26× magnification of lightsheet microscopy. Scale bar, 1 mm. (C) Representative 3D-projection images (coronal view) of the brainstem at 2.5× magnification of lightsheet microscopy. NTS and DMV regions are denoted. Scale bar, 1 mm (D) Representative optical-section images (coronal view) of NTS and DMV at 12.6× magnification of lightsheet microscopy. Scale bar, 100 μm.

The mouse brains processed through this protocol are almost transparent (Figure 1A), essential for the high quality of lightsheet fluorescence imaging. Consistent with our recent study,^1^ the 3D imaging of c-Fos protein expression shows that food intake can robustly trigger neuronal activities in the brain regions related to parasympathetic signals, e.g., the nucleus of the solitary tract (NTS) and the dorsal motor nucleus of the vagus (DMV) (Figures 1B–1D).

Of importance, this protocol is suitable for assessing neuronal activities in the mouse brains responding to diverse physiological or pathological stimuli, e.g., hormones, temperatures, cognitive functions, social interactions, or stresses. For instance, our previous work with the 3D imaging of c-Fos protein expression revealed the asymmetrical activation of the insular cortex in the mice challenged by intraperitoneal injection of lithium chloride.^6^ Therefore, the protocol can serve broad research purposes in neuroscience.

## Limitations

This protocol is readily applicable to intact, unsectioned mouse brains. All the utilized reagents are commonly available in biomedical research laboratories. However, a notable limitation of this protocol is its reliance on lightsheet microscopy (e.g., LaVision Biotec Ultramicroscope II). This demand for unique equipment may limit the adoption of this protocol in certain research settings.

## Troubleshooting

### Problem 1

Tissue distortion.

### Potential solutions

• Precooling the Decolorization Solution and the brain samples to 4°C minimizes excessive air bubble formation during the decolorization step, which may otherwise lead to tissue distortion (Step 7 and 8).
• Sodium azide prevents the incidental growth of bacteria or fungi during incubating at 37°C, which may otherwise damage tissue structures (Step 10, 11, 12 and 14).
• Precooling the 0.8% agarose to approximately 50°C before embedding the brain samples prevents heat-induced tissue distortion. (Step 17).

### Problem 2

Suboptimal quality of whole-tissue immunolabeling.

### Potential solutions

• Harvest the brain samples as quickly as possible to prevent tissue desiccation. If necessary, perform the tissue harvesting process submerged in 1× PBS. Nonspecific antibody binding may occur on the dried tissue surface. Desiccation also increases autofluorescence intensity, which may interfere with fluorescence imaging (Step 3).
• It is recommended to use the secondary antibodies conjugated with far-red fluorophores (e.g., Alexa Fluor 647), which provide a lower autofluorescence background in tissues (Step 14).
• Perform all the incubation steps with gentle rotation and ensure complete immersion of the brain samples during all the incubation steps.

### Problem 3

Suboptimal optical clearing and imaging quality.

### Potential solutions

• Prewarming the Perfusion Buffers to 37°C enables the efficient removal of erythrocytes, which contain high levels of light-absorbing hemoglobins that interfere with optical clearing (Step 2).
• After diluting the primary or secondary antibody into the Immunolabeling Buffer, filter the solution through a 0.22-μm syringe filter to remove any protein precipitates, which prevents the appearance of "speckles" in fluorescence imaging (Step 12 and 14).
• Remove any visible debris or contaminants from the brain samples before embedding, which may cause light scattering and interfere with fluorescence imaging (Step 16).
• Ensure complete dissolution of agarose in 1× PBS to prevent residual agarose particles, which compromises the quality of optical clearing (Step 17).

## Resource availability

### Lead contact

Further information and requests for resources and reagents should be directed to and will be fulfilled by the lead contact, Jing Yang (jing.yang@pku.edu.cn).

### Technical contact

Questions about the technical specifics of performing the protocol should be directed to the technical contact, Koukou Fu (2301110504@stu.pku.edu.cn).

### Materials availability

This study did not generate new unique reagents.

### Data and code availability

This study did not generate or analyze datasets. This study did not report original code.

## Acknowledgments

This work was supported by the National Natural Science Foundation of China (#32125017 and #82441057 to J.Y.).

## Author contributions

J.Y. conceived and designed the study. K.F. performed the experiments and analyzed the results. K.F. and J.Y. prepared the manuscript.

## Declaration of interests

The authors declare no competing interests.
